# Different Metabolic Roles for Alternative Oxidase in Leaves of Palustrine and Terrestrial Species

**DOI:** 10.3389/fpls.2021.752795

**Published:** 2021-11-04

**Authors:** Nestor Fernandez Del-Saz, Cyril Douthe, Marc Carriquí, Jose Ortíz, Carolina Sanhueza, Alicia Rivas-Medina, Allison McDonald, Alisdair R. Fernie, Miquel Ribas-Carbo, Jorge Gago, Igor Florez-Sarasa, Jaume Flexas

**Affiliations:** ^1^Laboratorio de Fisiología Vegetal, Departamento de Botánica, Facultad de Ciencias Naturales y Oceanográficas, Universidad de Concepción, Concepción, Chile; ^2^Research Group on Plant Biology Under Mediterranean Conditions, Departament de Biologia, Institute of Agro-Environmental Research and Water Economy, Universitat de les Illes Balears, Illes Balears, Spain; ^3^Departamento de Ingeniería Topográfica y Cartografía, Escuela Técnica Superior de Ingenieros en Topografía, Geodesia y Cartografía, Universidad Politécnica de Madrid, Madrid, Spain; ^4^Department of Biology, Wilfrid Laurier University, Waterloo, ON, Canada; ^5^Max-Planck-Institut für Molekulare Pflanzenphysiologie, Potsdam, Germany; ^6^Centre for Research in Agricultural Genomics (CRAG), CSIC-IRTA-UAB-UB, Barcelona, Spain; ^7^Institut de Recerca i Tecnología Agroalimentàries (IRTA), Edifici CRAG, Barcelona, Spain

**Keywords:** alternative oxidase pathway (AOP), cytochrome oxidase pathway (COP), electron partitioning to the AOP (τ_a_), primary metabolism, terrestrial species, palustrine species, heterophylly

## Abstract

The alternative oxidase pathway (AOP) is associated with excess energy dissipation in leaves of terrestrial plants. To address whether this association is less important in palustrine plants, we compared the role of AOP in balancing energy and carbon metabolism in palustrine and terrestrial environments by identifying metabolic relationships between primary carbon metabolites and AOP in each habitat. We measured oxygen isotope discrimination during respiration, gas exchange, and metabolite profiles in aerial leaves of ten fern and angiosperm species belonging to five families organized as pairs of palustrine and terrestrial species. We performed a partial least square model combined with variable importance for projection to reveal relationships between the electron partitioning to the AOP (τ_a_) and metabolite levels. Terrestrial plants showed higher values of net photosynthesis (A_N_) and τ_a_, together with stronger metabolic relationships between τ_a_ and sugars, important for water conservation. Palustrine plants showed relationships between τ_a_ and metabolites related to the shikimate pathway and the GABA shunt, to be important for heterophylly. Excess energy dissipation *via* AOX is less crucial in palustrine environments than on land. The basis of this difference resides in the contrasting photosynthetic performance observed in each environment, thus reinforcing the importance of AOP for photosynthesis.

## Introduction

Current life on Earth would not be possible without the evolution of biochemical processes that maintained energy entry in plants during land colonization (Delwiche and Cooper, 2015; De Vries et al., 2016; De Vries and Archibald, 2018; Gago et al., 2019). The earliest terrestrial plant ancestor, a charophycean alga, emerged from water approximately 500 million years ago (Bhattacharya and Medlin, 1998; Yoon et al., 2004; Harholt et al., 2016; Morris et al., 2018; Reski, 2018), undergoing physiological, structural, and biochemical changes to cope with the transition from an aqueous to a gaseous medium (Kenrick and Crane, 1997; Pires and Dolan, 2012; Vermeij, 2016). Among physiological and structural modifications from the first colonizing vascular land plants, specialized sexual organs, different kinds of leaves and roots, stomata, vascular and structural tissues allowed increases in plant size and water use efficiency (Kenrick et al., 2012; Assouline and Or, 2013; Proctor, 2014; Arteaga-Vazquez, 2016; Brodribb et al., 2020). At the biochemical level, changes in metabolic pathways favored the synthesis of phenolic compounds, lignin, plant hormones, isoprenes, heat shock proteins or superoxide dismutase to favor photosynthetic performance and plant growth under a highly stressful terrestrial environment (Lowry et al., 1980; Kenrick and Crane, 1997; Waters, 2003; Weng and Chapple, 2010; Bowman et al., 2017). As plant gas exchange involves water loss, survival in the dry atmosphere required that plants overcame desiccation forcing the first colonizing terrestrial plants to be close to sources of water, until new adaptations allowed their spread into the dry atmosphere of terrestrial habitats (Brodribb et al., 2020). In the meantime, the antioxidant systems were enhanced in land plants allowing them to survive several deleterious types of environmental stresses worldwide that induce oxidative stress and damage to the photosynthetic apparatus (Asada, 2006; Thomas et al., 2008; Gill and Tuteja, 2010; Zandalinas et al., 2021).

Currently, several metabolic pathways are identified as major energy-dissipating systems conferring metabolic adaptation in response to a large entry of sunlight energy in leaves (Niyogi, 1999; Raghavendra and Padmasree, 2003; Scheibe, 2004; Noguchi and Yoshida, 2008). Among these pathways, mitochondrial metabolism stands out for its interaction with photosynthesis, photorespiration and nitrogen assimilation (Raghavendra and Padmasree, 2003; Florez-Sarasa et al., 2016; O’Leary et al., 2020). In the mitochondrial electron transport system, oxygen consumption takes place simultaneously through the activities of cytochrome oxidase (COX) and alternative oxidase (AOX). Several studies in genetically engineered AOX-modified terrestrial model plants have suggested a role of AOX activity in optimizing photosynthesis under stress (Dahal and Vanlerberghe, 2018; Del-Saz et al., 2018a) by favoring the dissipation of excess energy and thus balancing cellular redox metabolism (Raghavendra and Padmasree, 2003; Del-Saz et al., 2018a; Vanlerberghe et al., 2020). In fact, there is *in vivo* evidence of a fine tuning of respiratory metabolism *via* AOX activity in leaves of crops and model terrestrial plant species exposed to abiotic stress as a mechanism to dissipate excess energy (Florez-Sarasa et al., 2012, 2016; Del-Saz et al., 2018a, b). Indeed, across the divergence of the plant kingdom, AOX is widespread and conserved, and it is of vital importance for plants (McDonald and Vanlerberghe, 2006; Del-Saz et al., 2018a; Selinski et al., 2018). Notably, AOX is hypothesized to have originated among anaerobic bacteria in an anoxic atmosphere, being important for redox homeostasis during the transition to an oxygen-rich atmosphere 2.45 billion years ago during the Great Oxidation Event (Moore et al., 2002; Finnegan et al., 2003; Catling and Claire, 2005).

Several clades that appeared during the diversification of terrestrial plants, which include bryophytes, ferns and angiosperms, returned to aquatic environments, necessitating physiological, structural and biochemical modifications (Robe and Griffiths, 2000; Rascio, 2002; Maberly, 2014). This transition from terrestrial to aquatic habitats occurred gradually with dynamic environmental changes that provided habitats in the palustrine wetland system and emergent heterophyllous amphibious plants, which are characterized by submerged and aerial leaves, and are precursors of the fully submerged habit (Maberly and Spence, 1989; Maberly, 2014). The fully submerged habit led many aquatic leaves to display metabolic adaptations to enhance carbon gain (Bowes and Salvucci, 1989; Keeley and Santamaría, 1992; Maberly and Madsen, 2002; Huang et al., 2020) and the aeration status to allow oxidative phosphorylation (Gibbs and Greenway, 2003). It is unknown whether the transition from land to the amphibious condition involved respiratory and metabolic adjustments when oxygen was not a limiting factor. Such adjustments could have happened due to the contrasting redox conditions that characterize both environments. Terrestrial plants are less often shaded by canopy trees and more often exposed to drought events (Valladares and Niinemets, 2007; Schlesinger and Bernhardt, 2020), resulting in vegetation adapted to both different sunlight energy input and soil water conditions. Indeed, variation in vegetation type is more affected by climate in terrestrial habitats than in palustrine habitats (Schlesinger and Bernhardt, 2020), which may support our idea of higher potential risks for redox balance in terrestrial habitats. With this in mind, comparisons of respiratory metabolism in terrestrial vascular plants and their close amphibian relatives could provide clues to different metabolic routes important for the leaf biochemistry in each ecosystem under aerobic conditions. These comparisons could be performed in leaves of amphibious plants because part of their foliage photosynthesizes and respires in the same gaseous medium as leaves of terrestrial plants. In this sense, the combination of “omics” technologies together with measurements of photosynthesis and respiration is optimal for further understanding of the metabolic regulation of plant physiological processes under different environmental conditions (Florez-Sarasa et al., 2012, 2016, 2019; Del-Saz et al., 2016; Flexas and Gago, 2018; Clemente-Moreno et al., 2019).

No previous study has evaluated the *in vivo* respiratory activities in ferns and palustrine angiosperms. In the present study, we compared ten species of ferns and angiosperms organized as pairs of palustrine and terrestrial species (from the same family). The *in vivo* respiratory activities, photosynthesis, and metabolite profiling of aerial leaves were determined using the oxygen isotope discrimination technique, leaf gas exchange and gas chromatography coupled to mass spectrometry (GC-MS), respectively. Further, to outline the climatic space occupied by these species, we overlapped values of mean annual temperature (MAT) and annual precipitation with Whittaker’s biomes classification (Whittaker, 1970; Wright et al., 2004). The main objective was to assess respiratory differences between terrestrial and palustrine plant species. In addition, relationships between metabolic routes and the AOX pathway were identified given their importance for leaf biochemistry in terrestrial and palustrine environments. We hypothesize that in terrestrial plants, these relationships could be important for the regulation of water conservation and redox state; whilst in palustrine plants, these relationships could be important for non-stress roles related to the adaptation to intermediate habitats between land and water (e.g., heterophylly).

## Materials and Methods

### Plant Material and Experimental Design

We selected five families of vascular plants, which consisted of one terrestrial species and its palustrine counterpart: (1) *Acanthus mollis* L. and *Hygrophila stricta* (Vahl) L. in Acanthaceae (angiosperm); (2) *Arum italicum* Mill. and *Anubias heterophylla* Engl. in Araceae (angiosperm); (3) *Trachelium caeruleum* L. and *Lobelia cardinalis* L. in Campanulaceae (angiosperm); (4) *Polypodium cambricum* L. and *Leptochilus pteropus* (Blume) Fraser-Jenk, in Polypodiaceae (fern); and (5) *Pteris vittata* L. and *Ceratopteris thalictroides* L. (Brongn) in Pteridaceae (fern) (Table 1). In the middle of autumn, terrestrial plant species were collected in the field with their underlying substrate (soil) at various coordinates in Mallorca (Spain; Table 1), and placed in plastic bags to be immediately transported to the University of Balearic Islands (Mallorca) where they were transplanted into plastic pots, using a sterile soil–peat mixture (3: 1 v/v). Then, the pots were maintained in a growth chamber under controlled conditions of 25°C, moderate light intensity of 350 μmol m^–2^ s^–1^ of photosynthetic photon flux density (PPFD), relative humidity above 40%, 12 h photoperiod, and watered to full soil capacity every 3–4 days. At the same time, commercial amphibious plants were distributed inside the same growth chamber as the terrestrial plants in different 34 × 45 cm water-tanks containing 20 ± 5 cm water-level, rooted in gravel/substrate for aquarium plants, and maintained under a moderate irradiance of 100 μmol m^–2^ s^–1^, according to the low light demand required for growing aquarium species as described in previous studies (Mommer et al., 2005; Koga et al., 2020). Four to six plants per terrestrial and palustrine species were maintained under different availability of light energy and water in each habitat. By doing this, we generated contrasting redox environments according to their different predominance in biomes with contrasting canopy openness and water availability as outlined in next subsection. All plants developed aerial leaves under growth chamber conditions until the beginning of experiments in the middle of winter. The upper-most fully expanded aerial leaves of all species were used for gas exchange, *in vivo* respiration, and metabolic profiling analyses.

**TABLE 1:** Classification, collection, and life histories of the different plant species used in this study.

**Family**	**Habitat**	**Plant species**	**Life span**	**Description**	**GPS Coordinates**
Acanthaceae	Palustrine	*Hygrophila stricta*	Perennial	Angiosperm that reaches a height of 70 cm tall with lance-shaped shade leaves that can be up to 10–15 cm long and 2 cm wide	—–
	Terrestrial	*Acanthus mollis*	Perennial	Clump-forming angiosperm that reaches a maximum 180 cm in height with obovate leaves up to 40 cm long and 25 cm wide	39°45′34.2″N 2°42′39.5″E
Araceae	Palustrine	*Anubias heterophylla*	Perennial	Rhizomatous angiosperm that reaches 30 cm tall in height and develops oval shade leaves that can be up to 38 cm long and 13 cm wide	—–
	Terrestrial	*Arum italicum*	Perennial	Herbaceous angiosperm that reaches 30 cm tall in height with arrow-shaped 20–30 cm long leaves	39°45′34.2″N 2°42′39.5″E
Campanulaceae	Palustrine	*Lobelia cardinalis*	Perennial	Herbaceous angiosperm that grows up to 1.2 m tall in height with coarsely toothed shade leaves over 15 cm long and 4 cm wide	—–
	Terrestrial	*Trachelium caeruleum*	Perennial	Herbaceous angiosperm that grows 0.5–1 m tall with small lance-shaped leaves over 7.5–10 cm long	39°45′34.2″N 2°42′39.5″E
Polypodiaceae	Palustrine	*Leptochilus pteropus*	Perennial	Rhizomatous fern that reaches 15–30 cm tall in height with narrow and twisted shade leaves that can be up to 20 cm long	—–
	Terrestrial	*Polypodium cambricum*	Perennial	Rhizomatous fern that grows 60 cm tall with fronds over 5–30 cm in length	39°47′26.3″N 2°41′23.3″E
Pteridaceae	Palustrine	*Ceratopteris thalictroides*	Annual	Shade-adapted rhizomatous fern that grows 15–30 cm high and 10–20 cm wide with finely branched leaves	—–
	Terrestrial	*Pteris vittata*	Perennial	Rhizomatous fern that grows up to 1 m and with fronds that are from 30 to 80 cm long	39°45′51.3″N 2°42′33.6″E

*Note that amphibious species were obtained from commercial sources in Mallorca (Spain).*

### Species Spatial Distribution

In order to assess the abundance of both terrestrial and palustrine plant species in locations and biomes with different environmental conditions, we studied the spatial distribution of these species considering data of MAT and mean annual precipitation (MAP) from the years 1980 to 2010. Different numbers of records among species were obtained from GBIF (Global Biodiversity Information Facility^1^): *A. italicum* (32875), *P. cambricum* (17980), *L. cardinalis* (5375), *P. vittata* (3906), *A. mollis* (2628), *T. caeruleum* (2559), *C. thalictroides* (2037), *L. pteropus* (196), *A. heterophylla* (55), and *H. stricta* (7). For greater accuracy, we increased the number of records of palustrine plants in Araceae and Acanthaceae, by substituting *Higrophylla stricta* (7) for *Higrophylla ringens* (1264) and *Anubias heterophylla* (55) for *Anubias* spp. Schott. (617) because of their similar distribution records (Supplementary Figure 1). Then, a random selection of records equalized the number of samples in each family and habitat; 2000 in Campanulaceae; 1500 in Pteridaceae; 1000 in Acanthaceae; 600 in Araceae, and 150 in Polypodiaceae. Finally, the spatial distribution of records randomly selected was studied with QGIS, a GIS software that combines species occurrences from GBIF with climate layers from WorldClim^2^. QGIS rasterized species occurrences and extracted MAT and MAP data across all grid cells of the species occurrence region, at a spatial resolution of 30 arc-seconds (∼1 km). Then, species classification into biomes was performed from a Whittaker diagram of MAT and MAP (Wright et al., 2004).

### Leaf Gas Exchange Measurements

Leaf gas exchange with Chl*a* fluorescence measurements were recorded every day from 10 am to 2 pm during the last 2 weeks of the experiment with an open infrared gas-exchange analyzer system (Li-6400; Li-Cor Inc., Lincoln, NE, United States) equipped with a leaf chamber fluorometer (Li-6400-40, Li-Cor Inc.) using aerial leaves of terrestrial and amphibious plants under light-saturating photosynthetic photon flux density (PPFD) of 1000 and 400 μmol m^–2^ s^–1^, respectively (to avoid photodamage as a consequence of a high PPFD), with 10% blue light, a vapor pressure deficit (VPD) of 1.35 ± 0.32 kPa, a CO_2_ concentration (C_a_) of 400 μmol CO_2_ mol^–1^, and 25°C air temperature. Net photosynthesis (A_N_) and stomatal conductance (*g*_s_) were determined after a steady state was reached (after c. 20 min). Once the gas exchange stabilized, five readings were taken in four to six plants per species, and averaged to be considered as the mean of the measured plant. Intrinsic WUEi was calculated as the ratio between A_N_ and *g*_s_. After a minimum 30 min under dark conditions, leaf dark respiration (*R*_dark_) was measured in three to five plants per species with at least five readings per plant, and estimations of leaf carbon balance were obtained from the ratio of *R*_dark_ to A_N_.

The quantum efficiency of the photosystem II (PSII)-driven electron transport was determined using the equation ΦPSII = (*F*_m_′ - *F*_s_)/*F*_m_′, where *F*_s_ is the steady-state fluorescence in the light (PPFD = 1000 and 400 μmol quanta m^–2^ s^–1^ for terrestrial and palustrine plants, respectively) and *F*_m_′ is the maximum fluorescence obtained with a light-saturating pulse (8000 μmol quanta m^–2^ s^–1^). The electron transport rate (ETR) was calculated as ETR = ΦPSII × PPFD × αβ, where α is the leaf absorptance, assumed to be 0.84, and β is the distribution of absorbed energy between the two photosystems, assumed to be 0.5 (Gallé and Flexas, 2010). At least five readings in two to four plants per species were taken and averaged to be considered as ETR values of the measured plant. The average ETR value for each species was used for estimations of the ratio of ETR to A_N_.

### Respiration and Oxygen-Isotope Fractionation Measurements

For respiratory measurements, the aerial leaves of terrestrial and palustrine plants were harvested and cut into pieces after 30 min in darkness to be placed in a 3 ml stainless-steel closed cuvette maintained at a constant temperature of 25°C. Air samples were sequentially removed from the cuvette and fed into the mass spectrometer (Delta XPlus; Thermo LCC, Bremen, Germany). Changes in the ^18^O/^16^O ratios and O_2_ concentration were obtained to calculate the oxygen-isotope fractionation and the electron partitioning to the AOP (τ_a_), allowing calculations of the *in vivo* activities of AOP and cytochrome oxidase pathway (COP) as described in Del-Saz et al. (2017a). Both end point fractionation values of the AOP (Δ_a_) and the capacity of the alternative pathway (*V*_alt_) were determined in leaves of terrestrial and palustrine plants treated with a solution of 10 mM potassium cyanide (KCN) for 30 min. For land plants, Δ_a_ values (*n* = 3) of 29.9 ± 0.2‰, 30.0 ± 0.2‰, 30.2 ± 0.5‰, 30.6 ± 0.2‰ and 30.3 ± 0.4‰ were obtained for *P. cambricum*, *P. vittata*, *A. italicum*, *A. mollis*, and *T. caeruleum*, respectively. For palustrine plants, Δ_a_ values of 32.5 ± 0.3‰, 30.8 ± 0.3‰, 31.2 ± 0.8‰, 31.4 ± 0.1‰, and 29.6 ± 0.2‰ were obtained for *A. heterophylla*, *C. thalictroides*, *H. stricta*, *L. cardinalis*, and *L. pteropus*, respectively. On the other hand, an assumed value of 20.0‰ for the end point fractionation values of the COP (Δ_c_) was used for the electron partitioning calculations as this has been shown to be fairly constant in most of the leaves and species examined (Ribas-Carbó et al., 2005). Total mitochondrial ATP production (*ATP*_total_) together with ATP production *via* COP (*ATP*_cop_) and AOP (*ATP*_aop_) were modeled from the activities of the COP and AOP of each measurement, assuming that electron flow through the AOP drives the synthesis of 11 ATP for each 6 O_2_ consumed whilst 29 ATP are formed for each 6 O_2_ consumed *via* COP (Del-Saz et al., 2017b). Values presented are the mean of six to eight measurements performed in four to six plants per species that were performed from 9 am to 6 pm on the same days as gas exchange measurements were performed during the last 2 weeks of the experiment. In addition, the engagement of AOP (ρ) was calculated as a percentage of the ratio of the *in vivo* activity of AOP (*v*_alt_) to *V*_alt_.

### Metabolite Profiling

Terrestrial leaves of palustrine and terrestrial plants were simultaneously sampled after 30 min in darkness on the last day of the experimental period, immediately frozen in liquid nitrogen, and stored at –80°C until further analysis. Metabolite extractions, derivatization and gas chromatography time of flight-mass spectrometry (GC-TOF-MS) analyses were carried out as previously described (Lisec et al., 2006). The GC-TOF-MS system was composed of a CTC CombiPAL autosampler, an Agilent 6890N gas chromatograph, and a LECO Pegasus III time-of-flight mass spectrometer running in EI + mode. Metabolites were identified by comparison with database entries of standards (Kopka et al., 2005; Schauer et al., 2005). The data of each terrestrial species were normalized to the mean of its respective palustrine counterpart (i.e., the value of all metabolites for each palustrine species was set to 1). The data represent averages of three to six measurements corresponding to material harvested from three to six individual plants per species.

### Statistical Analysis

Data of A_N_, WUEi, total respiration (*V*_t_), *in vivo* activity of COP (*v*_cyt_), *ATP*_cop_, and *ATP*_total_, were log-transformed to meet homoscedasticity. A two-way analysis of variance (*p* < 0.05) was performed with habitat level (terrestrial, palustrine) and plant family (Acanthaceae, Araceae, Campanulaceae, Polypodiaceae, and Pteridaceae) as fixed factors (Table 2), and Tukey’s *post hoc* test (*p* < 0.05) was used to determine differences in each respiratory and photosynthetic parameter between species (Figures 2, 3, Tables 3, 4, and Supplementary Tables 2, 3). Student’s *t*-tests were used for statistical analyses in Table 5 in order to compare data from terrestrial species with data from the respective palustrine counterpart in each family. To generate individual fold change data from the physiological parameters, we normalized each measurement of the terrestrial counterpart to the mean of the respective palustrine species, as for the GC-MS metabolite analyses, and Pearson coefficients were obtained with JMP^®^, Version 12.1.0 (SAS Institute Inc., Cary, NC, United States, 1989–2007; Table 6). Associations between the respiratory parameters and the metabolite profile were explored by applying the Partial Least Square (PLS) sparse regression as defined previously (Saccenti et al., 2014). Missing data in the metabolome dataset were imputed by employing a random forest imputation method before PLS analysis (Gromski et al., 2014). The “*pls*” package in R software was used to develop the PLS regression analysis. Also, this package includes a function to implement the variable importance for the projection (VIP) for single-response orthogonal score *plsr* models (Wehrens and Mevik, 2007).

**TABLE 2:** Significance of sources of variation after two-way analysis of variance analyses for each parameter.

	**Habitat**	**Family**	**Habitat × Family**
**ETR**	***	**	ns
**A_N_**	***	***	ns
***g*_s_**	ns	***	**
***R*_dark_**	ns	ns	*
**WUEi**	***	***	***
***V*_t_**	ns	***	***
***τ_*a*_***	***	***	***
***v*_cyt_**	**	***	***
***v*_alt_**	ns	***	***
***V*_alt_**	*	*	***
***ATP*_cop_**	**	***	***
***ATP*_aop_**	ns	***	***
***ATP*_total_**	ns	***	***

The sources of variance were Habitat, Family, and their interaction (Habitat × Family). ns, not significant effect. *p < 0.05; **p < 0.01; ***p < 0.001.

**FIGURE 1:**
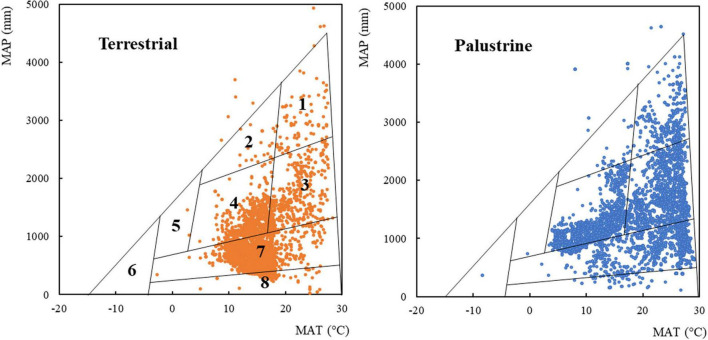
The boundaries of global biome type in relation to the climate factors mean annual temperature (MAT) and mean annual precipitation (MAP; Whittaker, 1970; Wright et al., 2004). For each habitat (terrestrial and palustrine), 5250 plant records (randomly selected and equalized, see section “Materials and Methods”) are overlaid on the climate envelopes of Whittaker’s biomes. Terrestrial and palustrine records are represented as brown and blue dots, respectively. (1) Tropical rainforest; (2) temperate rainforest; (3) tropical seasonal forest; (4) temperate forest; (5) boreal forest; (6) tundra; (7) woodland, shrubland, and grassland; (8) desert. Biome boundaries are only approximate. Specific abundances in each type of biome can be found in Supplementary Table 1.

**FIGURE 2:**
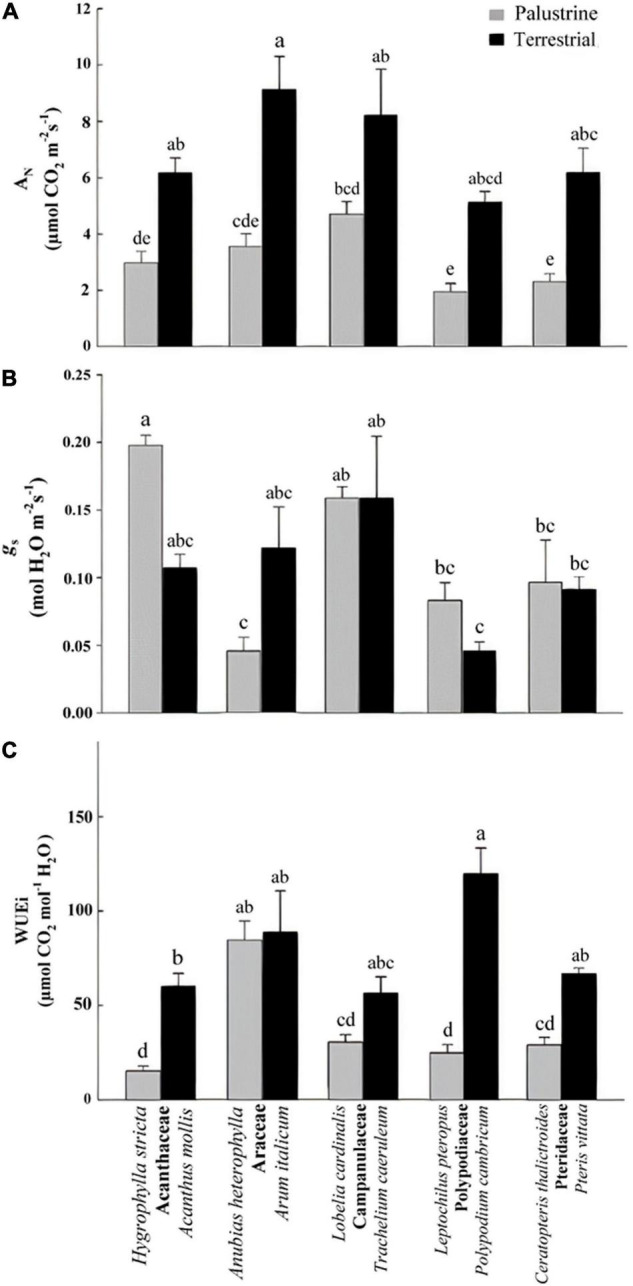
**(A)** Net photosynthesis (A_N_), **(B)** stomatal conductance (*g*_s_), and **(C)** intrinsic water-use efficiency (WUEi) in all palustrine and terrestrial species tested in this study. In **(C)**, values were calculated from mean values of A_N_ and *g*_s_. Four to six plants were used to characterize each species. Different letters indicate significant differences with a *p*-value < 0.05 determined by a *post hoc* Tukey–Kramer’s test.

**FIGURE 3:**
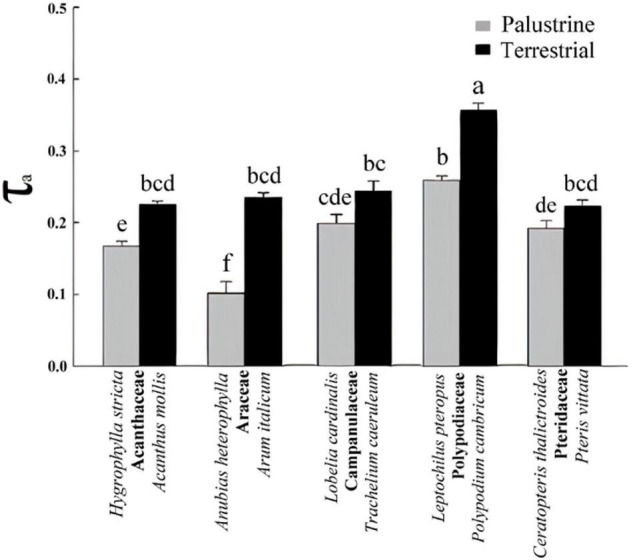
Electron partitioning to the alternative pathway (τ_a_) in all palustrine and terrestrial species tested in this study. Values are the mean of six to eight measurements obtained from 4 to 6 plants per species. Different letters indicate significant differences with a *p*-value < 0.05 determined by a *post hoc* Tukey–Kramer’s test.

**TABLE 3:** General characteristics of the studied terrestrial and palustrine plant species: the ratio of electron transport rate (ETR) to net photosynthesis (*A*_N_), the ratio of dark respiration (*R*_dark_) to *A*_N_, and the ratio of *v*_alt_ to *V*_alt_ (ρ).

**Family**	**Habitat**	**Plant species**	**ETR*/A*_N_**	***R*_dark_ */A*_N_**	**ρ *(%)***
Acanthaceae	Palustrine	*Hygrophila stricta*	8.58	0.190	57
	Terrestrial	*Acanthus mollis*	6.56	0.086	11
Araceae	Palustrine	*Anubias heterophylla*	8.49	0.124	9
	Terrestrial	*Arum italicum*	5.67	0.110	12
Campanulaceae	Palustrine	*Lobelia cardinalis*	9.79	0.135	14
	Terrestrial	*Trachelium caeruleum*	7.93	0.057	19
Polypodiaceae	Palustrine	*Leptochilus pteropus*	8.14	0.158	23
	Terrestrial	*Polypodium cambricum*	9.57	0.094	33
Pteridaceae	Palustrine	*Ceratopteris thalictroides*	11.27	0.256	24
	Terrestrial	*Pteris vittata*	10.77	0.088	22

**TABLE 4:** Total respiration (*V*_t_) and the *in vivo* activities of cytochrome oxidase (*v*_cyt_) and alternative oxidase (*v*_alt_) in aerial leaves of ten different terrestrial and palustrine plant species (see section “Materials and Methods”).

**Family**	**Habitat**	**Plant species**	***V*_t_ (nmol O_2_ g^–1^DW)**	***v*_cyt_ (nmol O_2_ g^–1^DW)**	***v*_alt_ (nmol O_2_ g^–1^DW)**
Acanthaceae	Palustrine	*Hygrophila stricta*	12.84 ± 2.12 **ab**	10.74 ± 1.83 **abc**	2.10 ± 0.298 **bc**
	Terrestrial	*Acanthus mollis*	15.17 ± 1.45 **a**	11.74 ± 1.09 **ab**	3.43 ± 0.373 **ab**
Araceae	Palustrine	*Anubias heterophylla*	7.03 ± 0.483 **cd**	6.34 ± 0.484 **cd**	0.694 ± 0.109 **d**
	Terrestrial	*Arum italicum*	11.77 ± 0.975 **ab**	9.00 ± 0.724 **bcd**	2.78 ± 0.264 **abc**
Campanulaceae	Palustrine	*Lobelia cardinalis*	15.39 ± 1.51 **a**	12.35 ± 1.54 **ab**	3.05 ± 0.419 **abc**
	Terrestrial	*Trachelium caeruleum*	14.86 ± 0.896 **a**	11.24 ± 0.708 **ab**	3.63 ± 0.310 **a**
Polypodiaceae	Palustrine	*Leptochilus pteropus*	8.37 ± 0.820 **bc**	6.21 ± 0.619 **d**	2.16 ± 0.210 **bc**
	Terrestrial	*Polypodium cambricum*	5.19 ± 0.559 **d**	3.33 ± 0.350 **e**	1.86 ± 0.222 **cd**
Pteridaceae	Palustrine	*Ceratopteris thalictroides*	20.03 ± 2.67 **a**	16.31 ± 2.35 **a**	3.71 ± 0.331 **a**
	Terrestrial	*Pteris vittata*	8.29 ± 0.760 **bcd**	6.44 ± 0.579 **cd**	1.84 ± 0.198 **cd**

*Values are the mean of six to eight measurements obtained from 4 to 6 plants per species. Different letters indicate significant differences with a *p-*value < 0.05 determined by *post hoc* Tukey–Kramer’s test.*

**TABLE 5:** Relative metabolite levels in leaves of 10 terrestrial and palustrine plant species belonging to five families of ferns and angiosperms as measured by GC-MS (see section “Materials and Methods”).

	**Acanthaceae**		**Araceae**		**Campanulaceae**		**Polypodiaceae**		**Pteridaceae**	
	***Hygrophila stricta***	***Acanthus mollis***	***Anubias heterophylla***	***Arum italicum***	***Lobelia cardinalis***	***Trachelium caeruleum***	***Leptochilus pteropus***	***Polypodium cambricum***	***Ceratopteris thalictroides***	***Pteris vittata***
***Amino acids***										
Alanine	1 ± 0.40	1.62 ± 0.30	1 ± 0.16	0.98 ± 0.47	1 ± 0.29	0.80 ± 0.22	**1 ± 0.32**	**0.09 ± 0.05**	1 ± 0.45	2.15 ± 0.65
Valine	1 ± 0.56	1.64 ± 0.32	**1 ± 0.14**	**4.76 ± 1.53**	1 ± 0.19	0.79 ± 0.29	1 ± 0.36	0.79 ± 0.28	1 ± 0.32	2.78 ± 1.07
Isoleucine	1 ± 0.40	1.27 ± 0.22	**1 ± 0.13**	**2.57 ± 0.41**	1 ± 0.39	0.67 ± 0.29	1 ± 0.32	0.96 ± 0.42	1 ± 0.30	3.25 ± 1.58
Glycine	1 ± 0.75	1.12 ± 0.57	**1 ± 0.19**	**0.32 ± 0.10**	nd		nd		1 ± 0.75	0.69 ± 0.23
Proline	**1 ± 0.38**	**5.35 ± 1.37**	**1 ± 0.13**	**0.26 ± 0.07**	**1 ± 0.27**	**0.33 ± 0.11**	1 ± 0.48	0.20 ± 0.13	**1 ± 0.42**	**2.83 ± 0.26**
Serine	1 ± 0.42	1.98 ± 0.25	1 ± 0.10	0.80 ± 0.30	1 ± 0.32	1.03 ± 0.39	**1 ± 0.19**	**0.37 ± 0.10**	1 ± 0.30	2.49 ± 0.80
Threonine	1 ± 0.38	0.46 ± 0.11	1 ± 0.50	0.43 ± 0.04	1 ± 0.28	0.54 ± 0.16	1 ± 0.15	0.53 ± 0.15	1 ± 0.33	1.47 ± 0.51
Phenylalanine	1 ± 0.42	0.56 ± 0.02	1 ± 0.49	0.99 ± 0.20	**1 ± 0.16**	**0.47 ± 0.13**	1 ± 0.30	0.51 ± 0.07	1 ± 0.25	1.77 ± 1.08
Asparagine	1 ± 0.36	1.85 ± 0.71	**1 ± 0.16**	**2.31 ± 0.05**	*1 ± 0.38	0.47 ± 0.01	**1 ± 0.12**	**0.01 ± 0.00**	1 ± 0.26	1.93 ± 0.80
Tryptophan	**1 ± 0.38**	**0.13 ± 0.02**	**1 ± 0.48**	**0.47 ± 0.11**	*1 ± 0.20	0.36 ± 0.00	1 ± 0.35	2.58 ± 0.80	1 ± 0.26	2.02 ± 1.14
Glutamic acid	**1 ± 0.39**	**9.04 ± 1.07**	**1 ± 0.12**	**1.97 ± 0.34**	1 ± 0.23	1.14 ± 0.34	1 ± 0.35	0.52 ± 0.12	**1 ± 0.52**	**4.08 ± 1.02**
***Organic acids***										
Glyceric acid	**1 ± 0.31**	**7.39 ± 1.90**	1 ± 0.20	1.88 ± 0.50	**1 ± 0.19**	**0.20 ± 0.04**	**1 ± 0.17**	**0.33 ± 0.13**	**1 ± 0.19**	**0.18 ± 0.04**
Pyruvic acid	1 ± 0.19	1.67 ± 0.39	nd		1 ± 0.21	0.60 ± 0.16	nd		*1 ± 0.27	0.24 ± 0.05
Citric acid	nd		1 ± 0.26	1.17 ± 0.45	nd		nd		**1 ± 0.29**	**6.59 ± 1.04**
Succinic acid	**1 ± 0.37**	**6.58 ± 0.79**	**1 ± 0.14**	**2.83 ± 0.55**	**1 ± 0.23**	**0.24 ± 0.02**	1 ± 0.18	1.19 ± 0.29	**1 ± 0.41**	**3.67 ± 0.19**
Fumaric acid	nd		1 ± 0.30	0.51 ± 0.08	1 ± 0.24	1.01 ± 0.49	1 ± 0.68	0.09 ± 0.03	1 ± 0.34	0.40 ± 0.03
Malic acid	nd		**1 ± 0.25**	**14.9 ± 3.56**	**1 ± 0.22**	**0.18 ± 0.05**	1 ± 0.28	0.60 ± 0.16	1 ± 0.24	1.05 ± 0.60
2-Oxoglutaric acid	**1 ± 0.19**	**46.9 ± 8.11**	**1 ± 0.19**	**0.30 ± 0.05**	*1 ± 0.27	0.29 ± 0.10	nd		1 ± 0.35	0.25 ± 0.05
Nicotinic acid	**1 ± 0.12**	**6.50 ± 1.95**	**1 ± 0.10**	**0.40 ± 0.08**	**1 ± 0.13**	**0.65 ± 0.07**	1 ± 0.33	0.63 ± 0.12	**1 ± 0.20**	**0.26 ± 0.03**
4-Aminobutyric acid	**1 ± 0.21**	**0.48 ± 0.05**	**1 ± 0.19**	**0.21 ± 0.06**	**1 ± 0.17**	**0.13 ± 0.04**	1 ± 0.66	0.49 ± 0.13	1 ± 0.43	1.90 ± 0.28
Threonic acid	1 ± 0.24	1.66 ± 0.32	**1 ± 0.19**	**14.6 ± 1.58**	**1 ± 0.22**	**0.27 ± 0.06**	1 ± 0.37	1.32 ± 0.41	**1 ± 0.25**	**10.5 ± 0.92**
***Antioxidants and secondary metabolism precursor***										
Quinic acid	**1 ± 0.37**	**0.09 ± 0.02**	**1 ± 0.19**	**0.37 ± 0.06**	1 ± 0.15	1.97 ± 0.17	1 ± 0.38	2.07 ± 0.28	**1 ± 0.10**	**166 ± 8.37**
Caffeoylquinic acid	**1 ± 0.27**	**0.01 ± 0.00**	nd		**1 ± 0.11**	**544 ± 77.8**	1 ± 0.16	1.37 ± 0.11	*1 ± 0.10	2.23 ± 0.24
Dehydroascorbic acid	1 ± 0.34	0.68 ± 0.08	**1 ± 0.14**	**1.70 ± 0.20**	1 ± 0.28	0.53 ± 0.04	**1 ± 0.18**	**20.2 ± 3.79**	**1 ± 0.40**	**45.6 ± 8.01**
Caffeic acid	1 ± 0.17	0.68 ± 0.11	1 ± 0.22	0.61 ± 0.15	1 ± 0.22	1.45 ± 0.26	1 ± 0.21	0.50 ± 0.03	1 ± 0.50	0.73 ± 0.04
***Sugars***										
Maltose	nd		**1 ± 0.33**	**8.55 ± 1.47**	nd		**1 ± 0.08**	**2.16 ± 0.30**	nd	
Rhamnose	1 ± 0.14	1.11 ± 0.22	**1 ± 0.22**	**6.33 ± 0.51**	**1 ± 0.13**	**2.47 ± 0.33**	nd		nd	
1,6-Anhydroglucose	**1 ± 0.20**	**0.28 ± 0.03**	1 ± 0.11	1.42 ± 0.23	**1 ± 0.16**	**13.5 ± 2.91**	**1 ± 0.28**	**4.70 ± 0.96**	1 ± 0.53	1.77 ± 0.45
Fructose	**1 ± 0.20**	**0.22 ± 0.05**	1 ± 0.07	1.05 ± 0.06	**1 ± 0.09**	**0.08 ± 0.00**	**1 ± 0.28**	**35.8 ± 5.56**	1 ± 0.23	1.00 ± 0.05
Glucose	**1 ± 0.29**	**8.35 ± 1.93**	1 ± 0.48	1.98 ± 0.76	**1 ± 0.29**	**0.04 ± 0.01**	**1 ± 0.38**	**183 ± 24.6**	**1 ± 0.64**	**26.5 ± 1.92**
Xylose	*1 ± 0.10	0.28 ± 0.05	**1 ± 0.31**	**2.05 ± 0.23**	**1 ± 0.03**	**0.53 ± 0.13**	nd		nd	
Sucrose	1 ± 0.26	1.92 ± 0.26	1 ± 0.16	1.14 ± 0.36	1 ± 0.23	0.91 ± 0.12	1 ± 0.37	1.17 ± 0.10	**1 ± 0.60**	**5.73 ± 0.39**
Raffinose	1 ± 0.23	1.69 ± 0.77	**1 ± 0.18**	**0.29 ± 0.04**	**1 ± 0.16**	**0.07 ± 0.01**	**1 ± 0.07**	**2.19 ± 0.33**	nd	
Trehalose	**1 ± 0.19**	**1.61 ± 0.06**	**1 ± 0.06**	**2.89 ± 0.37**	1 ± 0.14	2.24 ± 0.68	1 ± 0.33	1.66 ± 0.36	1 ± 0.78	0.33 ± 0.04
Melibiose	1 ± 0.19	1.53 ± 0.44	**1 ± 0.34**	**0.96 ± 0.02**	nd		nd		1 ± 0.53	2.10 ± 0.11
***Sugar-alcohols***										
Erythritol	1 ± 0.38	0.62 ± 0.08	**1 ± 0.19**	**8.21 ± 2.04**	**1 ± 0.13**	**1.64 ± 0.10**	nd		nd	
Galactinol	**1 ± 0.19**	**2.09 ± 0.09**	1 ± 0.23	1.50 ± 0.56	**1 ± 0.14**	**0.15 ± 0.01**	*1 ± 0.51	1.56 ± 0.56	1 ± 0.17	0.68 ± 0.14
Glycerol	1 ± 0.14	1.01 ± 0.25	1 ± 0.29	0.71 ± 0.10	**1 ± 0.16**	**2.53 ± 0.19**	**1 ± 0.21**	**0.33 ± 0.11**	1 ± 0.16	0.84 ± 0.16
Myo-inositol	**1 ± 0.21**	**2.18 ± 0.22**	**1 ± 0.22**	**19.6 ± 6.25**	**1 ± 0.05**	**1.35 ± 0.11**	**1 ± 0.13**	**0.20 ± 0.04**	**1 ± 0.42**	**25.8 ± 10.5**
*Other metabolites*										
Phosphoric acid	*1 ± 0.34	20.6 ± 8.45	**1 ± 0.17**	**0.18 ± 0.11**	1 ± 0.76	0.89 ± 0.25	1 ± 0.41	0.37 ± 0.23	1 ± 0.23	1.57 ± 0.78

Data represent averages of 3–6 measurements obtained from 3 to 6 plants per species, with significant differences in relative expression between terrestrial and palustrine plants per family in bold (*p-*value < 0.05). nd denotes primary metabolites in certain plant families that were not detected. *Denotes metabolites detected only in two replicates in palustrine or terrestrial species in certain families.

**TABLE 6:** Pearson correlation coefficients between fold changes in photosynthetic parameters levels (A_N_, g_s_, WUEi) and *in vivo* respiratory parameters levels (*V*_t_, *v*_cyt_, *τ_a,_ v*_alt_), and between fold changes in respiratory parameters (τ_a_ and *ATP*_total_) and ATP synthesis through each pathway (*ATP*_cop_ and *ATP*_aop_), in leaves of ten species of palustrine and terrestrial vascular plants (Table 1).

	**A_*N*_**	***g*_s_**	**WUEi**
***V*_t_**	–0.29	0.52	–0.45
***τ_a_***	**0.75**	0.23	0.27
***v*_cyt_**	–0.44	0.55	–0.55
***v*_alt_**	0.19	0.48	–0.17

	***ATP*_cop_**	***ATP*_aop_**	***τ_*a*_***

***ATP*_total_**	**0.98**	**0.87**	0.62*
***τ_*a*_***	0.43	**0.92**	−

Fold change values were log10 transformed and then used for the Pearson correlations. The same plants were used for all analyses, thus allowing 10-point correlations using the 4–6 replicates and the 10 species analyzed. The value in bold indicates a statistically significant Pearson coefficient with *p* value < 0.05. *Denotes a *p-*value = 0.058.

## Results

### Spatial Patterns

A species classification into biomes was obtained from a Whittaker diagram of MAT and MAP (Figure 1 and Supplementary Table 1; Wright et al., 2004). We observed species records in all biomes, especially in shrubland, temperate forest, tropical seasonal forest, woodland, and desert (25.6, 24.0, 22.2, 12.6, and 9.89% total records). A low register was found in tropical rainforest, grassland, temperate rainforest, boreal forest, and tundra (4.06, 1.11, 0.48, 0.03, and 0.02% total records). In general, palustrine species were more abundant than terrestrial species in biomes with values of MAP ≥ 1000 mm, such as temperate forest (33.0% palustrine *vs.* 15.1% terrestrial), tropical seasonal forest (32.3% palustrine *vs.* 12.2% terrestrial), and tropical rainforest (6.40% palustrine *vs.* 1.72% terrestrial). In biomes with values of MAP ≤ 1000 mm, palustrine species were more abundant only in woodland (19.3% palustrine *vs.* 5.96% terrestrial), whilst terrestrial species were more abundant than palustrine species in arid biomes such as shrubland (46.4% terrestrial *vs.* 4.72% palustrine) and desert (17.6% terrestrial *vs.* 2.16% palustrine). Specific abundances in each type of biome can be found in Supplementary Table 1.

### Leaf Gas Exchange

Regarding net photosynthesis (A_N_), comparisons between groups showed no differences between angiosperms (Acanthaceae, Araceae, Campanulaceae) and ferns (Polypodiaceae, Pteridaceae) in terrestrial habitats; however among palustrine species, A_N_ was significantly lower in the two ferns species compared to the angiosperm *L. cardinalis* (Campanulaceae; Figure 2A). When comparing between counterparts in each family, A_N_ was significantly higher (by 2.5-fold) in terrestrial species of Acanthaceae, Araceae, Polypodiaceae, and Pteridaceae. Regarding *g*_s_ among terrestrial species, this parameter was significantly lower in the fern *P. cambricum* (Polypodiaceae) only when compared with the angiosperm *T. caeruleum* (Campanulaceae). Contrary to what was observed for A_N_, no differences were found in *g*_s_ when comparing between counterparts in each family (Figure 2B).

With regard to WUEi, no major differences were observed between ferns and angiosperms in terrestrial habitats; whilst among palustrine species, the two ferns species showed a significantly lower WUEi when compared to the angiosperm *A. heterophylla* (Araceae; Figure 2C). Very similar to the trends observed for A_N,_ WUEi was significantly higher (by 3.7-fold) in terrestrial counterparts of Acanthaceae, Polypodiaceae, and Pteridaceae, with the terrestrial fern *P. cambricum* (Polypodiaceae) showing the highest values of WUEi, and both the palustrine angiosperm *H. stricta* (Acanthaceae) and fern *L. pteropus* (Polypodiaceae) displaying the lowest values of WUEi (Figure 2C). On the other hand, palustrine plants showed higher averaged values of ETR/A_N_ (9.25) and *R*_dark_/A_N_ (0.173) than terrestrial plants (ETR/A_N_ = 8.10, *R*_dark_/A_N_ = 0.087) mainly because their small A_N_, and secondary, because the lack of major variations in *R*_dark_ and ETR (Tables 2, 3 and Supplementary Table 2).

### Respiration and Electron Partitioning to the Alternative Oxidase Pathway

A high heterogeneity was found in *V*_t_, *v*_cyt_, and *v*_alt_ among all species. Considering that most of *V*_t_ takes place *via* COX activity, a similar heterogeneity was found in *v*_cyt_ and *V*_t_, with both varying significantly by 3.3 and 2.7-fold, across species in the terrestrial and palustrine environments, respectively. Both *v*_alt_ and τ_a_ showed less variability than *v*_cyt_ and *V*_t_ across terrestrial species (2.0 and 1.6-fold, respectively). In palustrine environments, higher variability was found in *v*_alt_, differing significantly 5.4-fold across species, whilst τ_a_ showed similar variability to *v*_cyt_ and *V*_t_ (2.6-fold). When comparing between counterparts in each family, *V*_t_ was significantly higher in terrestrial counterparts of Araceae (by 1.7-fold), and in palustrine counterparts from both fern families, Polypodiaceae and Pteridaceae (by 1.6-fold and 2.4-fold respectively; Table 2), differing slightly from *v*_cyt_, which was no different in terrestrial counterparts of Araceae (Table 4). A different pattern was observed for *v*_alt_, which was significantly higher in the terrestrial counterpart of Araceae (4.0-fold) and in the palustrine counterpart of Pteridaceae (2.0-fold). A similar behavior was observed for ATP production modeled from *v*_cyt_ and *v*_alt_ (Supplementary Table 3). Regarding τ_a_, the terrestrial counterparts of Acanthaceae, Araceae, and Polypodiaceae showed significantly higher values than their palustrine counterparts, 1.4, 2.3, and 1.4-fold, respectively. It is worth mentioning that in Polypodiaceae, the two ferns showed the highest values of τ_a_ in each habitat (Figure 3). On the other hand, leaves of *H. stricta* showed the highest engagement of AOP (ρ) (57%) mainly because the low *V*_alt_, followed by leaves of plants in Polypodiaceae and Pteridaceae (25.5%) that showed variability in *V*_alt_ and *v*_alt_, and by leaves of plants in Campanulaceae and of terrestrial plants in Araceae and Acanthaceae (14%) that displayed large *V*_alt_. The palustrine *A. heterophylla* showed the lowest ρ (9%) because the low *v*_alt_ (Tables 3, 4 and Supplementary Table 2).

In order to better understand the changes in photosynthetic parameters driving the species-specific response of the respiratory parameters, fold changes of A_N_, *g*_s_ and WUEi values were correlated with fold changes of *V*_t_, τ_a_, *v*_cyt_, and *v*_alt_ as described in the statistical analyses section. The only significant correlation (*r* = 0.75) can be found between A_N_ and τ_a_. Similarly, to study whether AOP contributes significantly to ATP synthesis, fold changes of τ_a_ and *ATP*_total_ values were correlated with fold changes of τ_a_, *ATP*_cop_ and *ATP*_aop_. Significant correlations can be found between *ATP*_total_ and energy synthesis by each pathway (*ATP*_cop_ and *ATP*_aop_; *r* = 0.98 and 0.87), and between τ_a_ and *ATP*_aop_ (*r* = 0.98; Table 6).

### Relative Metabolite Levels

By using GC-MS-based metabolite profiling from the aerial leaves of palustrine and terrestrial plants, we annotated 40 metabolites (Supplementary Table 5), including sugars, amino acids, organic acids, antioxidants and secondary metabolite precursors, as well as sugar-alcohols (Table 5). Although the identification of 17 metabolites (glycine, asparagine, tryptophan, phosphoric acid, pyruvic acid, citric acid, malic acid, fumaric acid, 2-oxoglutaric acid, quinic acid caffeoyl, maltose, rhamnose, xylose, raffinose, melibiose, erythritol, and galactinol) were only partly detected (*n* = 2) or not detected at all (nd) in certain species, they were considered for a general interpretation of the results. Significant changes (Student’s *t* test, *p* < 0.05) in metabolite levels were observed for each metabolite, in the comparison between terrestrial and palustrine counterparts in each family, with the exception of threonine, pyruvic acid, fumaric acid, and caffeic acid.

Focusing on photosynthetic routes, we observed that Campanulaceae, the only family which showed no significant differences in A_N_ between palustrine and terrestrial counterparts, showed the largest number of metabolites (19), mainly sugars and organic acids, with reduced levels in the terrestrial species when compared to the palustrine counterpart (Table 5). In contrast, terrestrial species of Acanthaceae, Araceae, Polypodiaceae, and Pteridaceae, with higher values of A_N_ than their palustrine counterparts, showed higher levels of sugars such as sucrose, fructose or glucose (Table 5), suggesting a higher energy status. We also observed that Araceae, with significantly higher *g*_s_ in the terrestrial counterpart, was the only family also showing higher levels of metabolites such as malate and maltose, which are considered of interest due to their roles in determining stomatal movement (Fernie and Martinoia, 2009; Araújo et al., 2011; Gago et al., 2016).

Regarding respiratory routes, in Araceae, the only family showing higher *V*_t_ in the terrestrial counterpart, the lack of change and decrease in citrate and 2-oxoglutarate levels, respectively, together with increases in downstream intermediates (succinate and malate) suggests a high TCA cycle activity (Table 5). This pattern was significantly different (increased citrate levels with no changes in 2-oxoglutarate and malate) in the two terrestrial fern species that displayed lower *V*_t_ and *v*_cyt_, when compared to their palustrine counterparts, presumably due to lower TCA cycle decarboxylation activity. In this comparison, pronounced differences in γ-aminobutyric acid (GABA) levels – which are intimately connected to TCA cycle activity – between ferns and angiosperms suggest a different role for the GABA-shunt. In addition, the large accumulation of sugars such as sucrose, glucose, and fructose in ferns (Table 4) coincided with an accumulation of antioxidant and secondary metabolism precursors such as quinic acid and dehydroascorbic acid, likely indicative of a reduction in sugar oxidation by glycolysis and the TCA cycle while also promoting the accumulation of antioxidant and secondary metabolism precursors (Table 5). Notably, in Araceae, the only family showing higher values of *v*_alt_ in the terrestrial counterpart, we observed higher levels of metabolites such as valine, isoleucine, and malate, which are considered of interest due to their positive correlation with *v*_alt_ in previous studies (Florez-Sarasa et al., 2012; Del-Saz et al., 2016).

**FIGURE 4:**
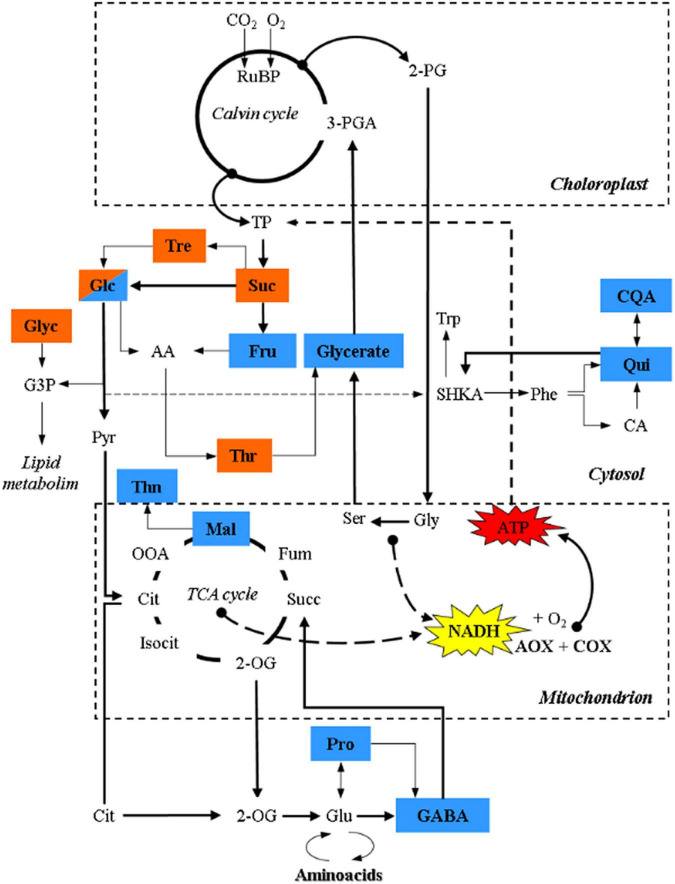
Schematic representation of the TCA cycle and its connection with metabolites, related to sugar metabolism, photorespiration and secondary metabolism, showing significant relationships with τ_a_ identified with a PLS approach through multivariate regression modeling. Brown and blue colors denote significant relationships with τ_a_ in terrestrial and palustrine environments, respectively. 2-PG, 2-Phosphoglycolate; 3-PGA, 3-Phosphoglyceric acid; RuBP, RuBisCO; TP, triose phosphate; Suc, sucrose; Tre, Trehalose; Glc, Glucose; AA, Ascorbic acid; Fru, fructose; Thr, threonic acid; Gly, glycine; Glyc, glycerol; G3P, glycerol 3-phosphate; SHKA, shikimate; Trp, tryptophan; Phe, phenylalanine; CA, caffeic acid; Qui, quinic acid; CQA, caffeoylquinic acid; Pyr, pyruvate; Cit, citrate; OOA, oxaloacetate; Mal, malate; Thn, threonine; Fum, fumarate; Suc, succinate; 2-OG, 2-oxoglutarate; Pro, proline; GABA, γ-aminobutyric acid; Glu, glutamate.

Given the observed general tendency of several physiological parameters to correlate with several metabolites (Figures 2A, 3 and Table 5), we further investigated the observed respiratory patterns for each habitat group employing PLS statistical modeling combined with variable importance for projection (VIP) as a criterion to elucidate metabolite relevance from the generated models (Gago et al., 2016). This modeling helps to highlight putative metabolic networks that differentially drive the respiratory processes in the terrestrial as compared to the palustrine species studied. We used *V*_t,_ *v*_cyt,_ *v*_alt_, and τ_a_ as response variables and, after cross-validation (CV) of the generated models by the PLS, only models for τ_a_ can be considered robust due to the display of a *R*^2^ higher than 0.6, for both terrestrial (*R*^2^ = 0.62) and palustrine (*R*^2^ = 0.7) habitats. For palustrine species, significant associations with phosphoric acid, proline, glucose, malic acid, glyceric acid, quinic acid, quinic acid caffeoyl, fructose, GABA, and threonine were observed (Figure 4 and Supplementary Table 4). For terrestrial species, associations with τ_a_ were observed for trehalose, sucrose, glucose, threonic acid and glycerol (Supplementary Table 4). Interestingly, sugar metabolism was importantly related to τ_a_ for both lifestyle strategies, glucose being the only metabolite significantly associated in both; despite sugar metabolism in each family differing in the other metabolite associations. Terrestrial species associated mostly with levels of trehalose and sucrose, while palustrine species were mainly associated with phosphoric acid and proline.

## Discussion

### Habitats Are Associated With Different A_N_, Water Use Efficiency and Electron Partitioning to Alternative Oxidase Pathway

In order to characterize terrestrial and palustrine species under the contrasting redox conditions that broadly differentiate both habitats, we decided to maintain plants under different light intensities to fall close to an optimum for each lifestyle. This is because palustrine plants are more often covered by dense canopy trees in humid forests than terrestrial plants in semi-arid Mediterranean forests, according to spatial distribution of plant records and sample collection coordinates of terrestrial plants (Figure 1 and Table 1). Besides, in humid forest, ground layer plant species may display shade adaptations like low light saturation and light compensation points (Chazdon and Pearcy, 1991; Meng et al., 2014), which led us to photosynthetically characterize these species at different PPFD. We did not expose plants to changing light intensities because it is well known that changes in growth light intensity does not affect oxygen isotope discrimination or τ_a_ as observed in leaves of *Arabidopsis thaliana* (Florez-Sarasa et al., 2011) and of sun and shade species (Noguchi et al., 2001). However, we ensured that experimental conditions were non-stressful, and enough to allow ETR/*A*_N_ values typical of irrigated plants, positive leaf carbon balance and low AOP engagement (and enough overcapacity) in all species (Table 3).

As leaves of terrestrial plants have large energy input because in air the light level is high, the terrestrial species *A. mollis*, *A. italicum*, *P. cambricum*, and *P. vittata* showed higher A_N_ than their palustrine counterparts *H. stricta*, *A. heterophylla*, *L. pteropus*, and *C. thalictroides* in Acanthaceae, Araceae, Polypodiaceae, and Pteridaceae, respectively (Figure 2A). This coincided with higher levels of sugars (e.g., sucrose, fructose, and glucose; Table 5), which were considered as markers of high photosynthetic activity (Gago et al., 2016). In contrast, no differences in A_N_ were found between *T. caeruleum* and *L. cardinalis* in Campanulaceae, which coincides with important reductions in sugars and organic acids in *T. caeruleum* with respect to *L. cardinalis* (Table 5). Because the higher A_N_, WUEi, the ratio between A_N_ and *g*_s_, was found to be larger in Acanthaceae, Polypodiaceae, and Pteridaceae (Figure 2C), which could be in line with previous studies describing a differential regulation of ecosystem (WUE) among biomes. In arid ecosystems, WUE is primarily controlled by evaporation; whilst in sub-humid regions, WUE is mostly regulated by assimilation (Yang R. et al., 2016), which could be partly due to a different predominance of palustrine and terrestrial records displaying contrasting values of WUEi (Figure 1 and Figure 2C) agreeing with the idea of water losses acting as a driving force for the evolution in land plants of gas exchange regulation system (Raven, 2002; Berry et al., 2010; Assouline and Or, 2013).

Contrary to A_N_, total respiration (*V*_t_) was not higher in the terrestrial species of Acanthaceae, Araceae, Polypodiaceae, and Pteridaceae than in their palustrine counterparts. Differences in *V*_t_ were found among families in each habitat and between ferns and angiosperms (Table 4), similar to previous studies (Choy-Sin and Suan, 1974; Boyce and Mohamed, 1987; Davey et al., 2004; Hilman and Angert, 2016; Zhu et al., 2021). Variability was also found regarding *v*_alt_ and *v*_cyt_ (Table 2). Respiration in leaves is highly variable among species as it depends on leaf characteristics such as leaf lifespan, nitrogen content, growth forms, and differential nutritional requirements, regardless of lifestyle or biome (Grime and Hunt, 1975; Reich et al., 1998; Lusk and Reich, 2000; Millenaar et al., 2001; Wright et al., 2004; Atkin et al., 2015). Moreover, the carbon cost for leaf growth and maintenance may differ among species (Lambers et al., 2008). This is why τ_a_, which represents the contribution of AOX to *V*_t_, represents a better proxy to evaluate the importance of AOX activity for plant respiration when comparing among different plant species. *In vivo* AOX activity accounted for 10-36% of *V*_t_ in both palustrine and terrestrial species considered here, which is within the range of values observed under both stressful and non-stressful conditions in terrestrial species (10–50%; Del-Saz et al., 2018a), and here, it was strongly influenced by habitat (Table 2). The contribution of AOX to *V*_t_ was significantly higher in terrestrial species from Acanthaceae, Araceae, and Polypodiaceae (Figure 3). In model terrestrial plants, previous studies reported τ_a_ increases under abiotic stressors mainly due to reductions in *v*_cyt_ because the COX pathway is more sensitive to stressors than the AOX pathway (Del-Saz et al., 2018a), which helps to explain the different effect of habitat on both *v*_cyt_ and *v*_alt_ (Table 2). Considering the highest values of A_N_ and τ_a_ observed among terrestrial species (Figures 2A, 3) and the significant Pearson coefficient between these parameters (Table 6), the AOP is likely more important for the dissipation of excess energy in terrestrial plants than in palustrine plants, which is in line with previous studies describing higher oxygen isotope discrimination in sun leaves than in shade leaves (Noguchi et al., 2001). Moreover, this coincided with metabolic increases in the levels of several sugars and A_N_ (Figure 2A and Table 5). Interestingly, τ_a_ was variable among terrestrial and palustrine species (Figure 3), suggesting that *v*_alt_ is coupled to fundamental metabolic processes under non-stress conditions that may differ among species (Florez-Sarasa et al., 2016). Regarding the differences observed between groups, previous studies suggested that the post-translational regulation of AOXs in ferns may differ from those of angiosperms because of the presence of a SerI residue instead of a CysI residue in the majority of the AOX protein sequences analyzed, which could presumably affect *v*_alt_ (Neimanis et al., 2013).

### The Electron Partitioning to the Alternative Oxidase Pathway Is Linked to Habitat-Specific Metabolic Routes

A PLS approach through multivariate regression modeling identified significant relationships only between τ_a_ and several metabolites in each habitat (Figure 4 and Supplementary Table 4). In terrestrial plants, significant relationships were identified only between τ_a_ and metabolites related to sugar metabolism (sucrose, glucose, and trehalose). All of these carbohydrates are closely linked to glycolytic activity or sucrose synthesis that are highly dependent on leaf ATP synthesis or requirements (Lunn et al., 2006; Dimroth and von Ballmoos, 2008; Lim et al., 2020). In addition, the accumulation of these sugars likely confers osmotolerance and redox homeostasis in both ecosystems (Robe and Griffiths, 2000). Sucrose is a metabolic precursor of trehalose, *via* trehalose-6-phosphate, which acts as a signal for high carbon availability in the form of sucrose (Schluepmann et al., 2004; Lunn et al., 2006; Paul et al., 2010; Fichtner and Lunn, 2021), which is in line with the high rates of A_N_ observed in terrestrial plants (Figure 2A). Trehalose is hydrolyzed by trehalase into glucose, and together with fructose (a product of the reactions catalyzed by both invertase and sucrose synthase) are metabolic precursors of ascorbic acid (AA), one of the most abundant antioxidants in plants (Smirnoff and Wheeler, 2000; Hossain et al., 2017). AA can be metabolized to compounds like threonate (Hancock and Viola, 2005; DeBolt et al., 2006; Smirnoff, 2018) which showed a significant relationship with τ_a_ in terrestrial plants. Notably, previous studies under salinity conditions highlighted a relationship between the AOP and erythronic acid (Del-Saz et al., 2016), a degradation product of AA (Green and Fry, 2005), reinforcing the role of the AOP in mitochondrial AA synthesis (Millar et al., 2003; Bartoli et al., 2006; Del-Saz et al., 2016). In addition, threonate is also a precursor of osmoprotectants (Guerrier et al., 2000; Jouve et al., 2004; Muscolo et al., 2015). On the other hand, τ_a_ in terrestrial plants also showed a significant relationship with glycerol, which is a lipid precursor, that similar to trehalose, is thought to be produced as a consequence of an enhanced CO_2_ assimilation in the Calvin-Benson cycle and/or from starch degradation (Liska et al., 2004), which corresponds to the highest values of photosynthesis, foliar carbon balance and oxygen isotope discrimination observed in terrestrial plants (Figures 1A, 3 and Table 3).

Palustrine plants displayed a higher energy efficiency of respiration bearing in mind their lower τ_a_, the significant Pearson coefficient between *ATP*_aop_ and *ATP*_total_ (Table 6), and the highest VIP value obtained from the relationship between τ_a_ and phosphate (Supplementary Table 4), perhaps indicative of a tendency to save phosphorus during oxidative phosphorylation for the benefit of ATP synthesis *via* COX. Besides, we identified relationships between τ_a_ and primary metabolites related to sugar metabolism, photorespiration, secondary metabolism, the TCA cycle and ammonium assimilation. Precisely, we found a significant relationship between τ_a_ and glycerate, corresponding to the described role of AOP in dissipating reducing equivalents from photorespiration (Watanabe et al., 2016; Timm and Hagemann, 2020), and suggesting a role of photorespiration in palustrine plants as previously described (Maberly and Spence, 1989). The relationships between τ_a_ and acyl-quinic acids (Qui, CQA; Figure 4) in palustrine plants suggest a participation of the AOP in modulating carbon supply for these chlorogenic acids, whose accumulation is associated with enhanced tolerance to oxidative stress (Tamagnone et al., 1998; Niggeweg et al., 2004), and competes with the accumulation of shikimate and derived metabolites (Marsh et al., 2009), such as phenylalanine and tryptophan. The reversible esterification of caffeoyl-CoA (whose metabolic precursor is CA) with Qui produces CQA. By the conversion of Qui to shikimate (Clifford et al., 2017), the shikimate pathway provides precursors for the synthesis of tryptophan that in turn is a metabolic precursor for the biosynthesis of auxins. In heterophyllous amphibious plants, auxin synthesis may be enhanced due to alterations in the perception of blue light in submerged leaves. This is part of a mechanism to coordinate, together with other plant hormones, phenotypic plasticity in leaf form or heterophylly (Nakayama et al., 2012, 2014, 2017; Li et al., 2019, 2021). On the other hand, the significant relationships between τ_a_ and malate, GABA, and proline suggest that the AOP could also be related to the carbon supply for both the TCA cycle and ammonium assimilation. Through the mitochondrial 2-OG/malate transporter, malate can facilitate GABA transport (Ramesh et al., 2018; Bown and Shelp, 2020), whose synthesis mainly occurs from glutamate by the cytosolic glutamate decarboxylase, alternatively through polyamine degradation (Yang Y. et al., 2016), or by the oxidation of proline to glutamate in mitochondria (Fait et al., 2008; Shelp et al., 2012). Moreover, both GABA and proline may act as osmoprotectants and their catabolism in mitochondria can provide reducing equivalents as substrates for the AOP (Studart-Guimarães et al., 2007; Michaeli et al., 2011; Florez-Sarasa et al., 2021), which is in agreement with the relationships identified between τ_a_ and these metabolites in palustrine plants (Figure 4 and Supplementary Table 4). On top of this, GABA can act as a transducer of environmental stress signals leading to the activation of genes for ethylene and abscisic acid biosynthesis (Kinnersley and Turano, 2000; Forde and Lea, 2007). Overall, the relationships between τ_a_ and metabolites related to hormone biosynthesis and signaling in palustrine environments could be especially relevant for heterophyllous amphibious plants. All these signaling metabolites, together with gibberellins, mediate perception and responses to fluctuations of water levels, and control the synthesis of new developing aerial leaves in the transition from a submerged to an aerial habit (Cox et al., 2004; Jackson, 2008; Chater et al., 2014; Kim et al., 2018). Whilst some evidence has suggested that plant hormones such as abscisic acid, ethylene, gibberellins, and auxins are part of signaling networks controlling AOX expression (Ivanova et al., 2014; Berkowitz et al., 2016), their control of *in vivo* AOX activity remains, even in model terrestrial plants, to be tested.

## Conclusion

Here we performed a comparative study of photosynthesis, WUEi, and respiration in palustrine and terrestrial species of angiosperms and ferns widely distributed across biomes, and maintained at different availability of energy and water in their habitats. Our experimental design does not allow the identification of the most important primary force (light or water) driving associations between the respiratory parameters and the metabolites. However, under different redox conditions that broadly characterize their habitats in nature, we found evidence of a large entry of energy into leaves of terrestrial plants considering their higher values of A_N_, WUEi, and τ_a_, as well as their significant relationships between τ_a_ and metabolites related to both sugar metabolism and osmotolerance. In palustrine plants, changes in τ_a_ could modulate the supply of carbon skeletons from sugars to metabolic routes involved in the production of hormones and signaling molecules important for heterophylly (e.g., the shikimate pathway and GABA shunt). Further experiments are needed in amphibious plants in order to study the precise regulation of the AOX pathway during the development of new aerial leaves during their emergence from water. In addition, the low τ_a_ observed together with the identification of τ_a_ relationships with phosphoric acid and other respiratory parameters suggests that mitochondrial electron partitioning contributes to maximizing the ATP yield of respiration in palustrine plants.

## Dedication

We would like to honor this manuscript to Prof. James N. Siedow. Jim taught me how to take science so seriously that only Duke basketball was at the same level. Jim could simultaneously smash you with the toughest question of the world, or plant biochemistry, and ensure that you could find the answer by yourself. The velocity of his brain was so high that by the time anyone could catch up with him, he was already smashing with the next joke. His jokes were always sharp, incisive, and funny. And, “so, What’s your point?” – MR-C.

## Data Availability Statement

The raw data supporting the conclusions of this article will be made available by the authors, without undue reservation.

## Author Contributions

JF, JG, MR-C, and ND-S conceived and designed the idea of this experiment. MC identified and recollected all plant species. CD carried out the gas-exchange measurements. ND-S carried out the measurements of respiration. IF-S carried out the metabolic analysis. JG carried out the PLS approach. AR-M carried out the spatial distribution analysis. ND-S, JO, and CS wrote the first draft of the manuscript with subsequent inputs from all co-authors. All authors have read and agreed to the published version of the manuscript.

## Conflict of Interest

The authors declare that the research was conducted in the absence of any commercial or financial relationships that could be construed as a potential conflict of interest.

## Publisher’s Note

All claims expressed in this article are solely those of the authors and do not necessarily represent those of their affiliated organizations, or those of the publisher, the editors and the reviewers. Any product that may be evaluated in this article, or claim that may be made by its manufacturer, is not guaranteed or endorsed by the publisher.

## Footnotes

1
http://www.gbif.org

2
https://www.worldclim.org
