# Supplementary material for: Different Metabolic Roles for Alternative Oxidase in Leaves of Palustrine and Terrestrial Species
Source: Front Plant Sci. 2021 Nov 4;12:752795. doi: 10.3389/fpls.2021.752795 (PMC8600120; doi:10.3389/fpls.2021.752795)
Supplement: Supplementary file 1 [file Table_1.docx]

**Supporting information Table 1.** Specific abundances in biomes of the distribution of randomly selected and equalized plant records (≈10500) from different terrestrial and palustrine species belonging to the five families of ferns and angiosperms evaluated in this study (see Material and Methods). For each species, abundances of records were obtained from GBIF (http://www.gbif.org) and overlaid on the climate envelopes of Whittaker’s biomes (Whittaker 1970; Wright *et al*., 2004). The number of selected records for each species is shown at the bottom of table. The percentage abundance of total, palustrine, and terrestrial records in each biome are shown on the right side of the table.

|  | **Acanthaceae** | | **Araceae** | | **Campanulaceae** | | **Polypodiaceae** | | **Pteridaceae** | |  |  |  |
| --- | --- | --- | --- | --- | --- | --- | --- | --- | --- | --- | --- | --- | --- |
|  | *Hygrophila ringens* | *Acanthus mollis* | *Anubias spp*. Schott | *Arum italicum* | *Lobelia cardinalis* | *Trachelium caeruleum* | *Leptochilus pteropus* | *Polypodium cambricum* | *Ceratopteris thalictroides* | *Pteris vittata* | Total records | Palustrine records | Terrestrial records |
| Tropical rainforest | 20 | 1 | 98 | 0 | 18 | 0 | 57 | 0 | 142 | 89 | 4.06% | 6.40% | 1.72% |
| Temperate rainforest | 5 | 2 | 0 | 0 | 0 | 0 | 1 | 0 | 20 | 22 | 0.48% | 0.50% | 0.46% |
| Tropical seasonal forest | 440 | 44 | 455 | 3 | 219 | 126 | 74 | 7 | 502 | 458 | 22.2% | 32.3% | 12.2% |
| Temperate forest | 120 | 178 | 2 | 103 | 1241 | 183 | 2 | 44 | 361 | 281 | 24.0% | 33.0% | 15.1% |
| Boreal forest | 0 | 0 | 0 | 0 | 1 | 1 | 0 | 1 | 0 | 0 | 0.03% | 0.02% | 0.04% |
| Tundra | 0 | 0 | 0 | 0 | 2 | 0 | 0 | 0 | 0 | 0 | 0.02% | 0.04% | 0.00% |
| Woodland | 386 | 29 | 40 | 8 | 150 | 67 | 12 | 0 | 419 | 208 | 12.6% | 19.3% | 5.96% |
| Shrubland | 15 | 686 | 0 | 477 | 221 | 919 | 0 | 88 | 11 | 259 | 25.6% | 4.72% | 46.4% |
| Grassland | 0 | 5 | 4 | 3 | 78 | 1 | 1 | 1 | 1 | 22 | 1.11% | 1.61% | 0.61% |
| Desert | 8 | 54 | 0 | 6 | 69 | 703 | 1 | 9 | 35 | 151 | 9.89% | 2.16% | 17.6% |
|  | 994 | 999 | 599 | 600 | 1999 | 2000 | 148 | 150 | 1491 | 1490 |  |  |  |
