# Supplementary material for: Different Metabolic Roles for Alternative Oxidase in Leaves of Palustrine and Terrestrial Species
Source: Front Plant Sci. 2021 Nov 4;12:752795. doi: 10.3389/fpls.2021.752795 (PMC8600120; doi:10.3389/fpls.2021.752795)
Supplement: Supplementary file 2 [file Table_2.docx]

**Supporting information Table 2.** Values of photosynthetic electron transport rate (ETR); respiration (*R*_dark_); and capacity of the alternative pathway (*V*_alt_) in aerial leaves of ten different terrestrial and palustrine plant species. Different letters indicate significant differences with a p value < 0.05 determined by post hoc Tukey–Kramer's test. Values are means ± SE for 2-4, 3-5, and 3 biological replicates for ETR, *R*_dark_, and *V*_alt_, respectively. * denotes data obtained only in two plants per species.

| **Family** | **Habitat** | **Plant species** | **ETR (µmols electrons m^-2^ s^-1^)** | ***R_dark_* (µmol CO_2_ m^-2^ s^-1^)** | ***V_alt_* (nmol O_2_ g^-1^ DWs^-1^)** |
| --- | --- | --- | --- | --- | --- |
| Acanthaceae | Palustrine | *Hygrophilla stricta* | 25.54 ± 2.67 **cd** | 0.566 ± 0.120 **ab** | 3.70 ± 0.131 **e** |
|  | Terrestrial | *Acanthus mollis* | 40.49 ± 7.25 **abcd** | 0.529 ± 0.171 **ab** | 30.90 ± 4.10 **a** |
|  |  |  |  |  |  |
| Araceae | Palustrine | *Anubias heterophylla* | 30.11 ± 3.93 **bcd** | 0.439 ± 0.126 **ab** | 7.42 ± 0.866 **de** |
|  | Terrestrial | *Arum italicum* | 51.71± 8.95 **ab** | 1.01 ± 0.191 **a** | 23.54 ± 5.83 **ab** |
|  |  |  |  |  |  |
| Campanulaceae | Palustrine | *Lobelia cardinalis* | *46.11 ± 3.88 **abcd** | 0.635 ± 0.098 **ab** | 21.40 ± 1.82 **abc** |
|  | Terrestrial | *Trachelium caeruleum* | 65.21 ± 7.49 **a** | 0.470 ± 0.054 **ab** | 19.37 ± 3.19 **abcd** |
|  |  |  |  |  |  |
| Polypodiaceae | Palustrine | *Leptochilus pteropus* | 15.78 ± 1.62 **d** | 0.307 ± 0.058 **b** | 9.37 ± 0.694 **cde** |
|  | Terrestrial | *Polypodium cambricum* | 49.16 ± 0.923 **abc** | 0.484 ± 0.097 **ab** | 5.72 ± 0.275 **de** |
|  |  |  |  |  |  |
| Pteridaceae | Palustrine | *Ceratopterys thalictroides* | 25.90 ± 1.67 **cd** | 0.634 ± 0.089 **ab** | 15.40 ± 3.07 **bcde** |
|  | Terrestrial | *Pteris vittata* | 66.55 ± 4.83 **a** | 0.542 ± 0.182 **ab** | 8.47 ± 1.69 **cde** |
