# Supplementary material for: Different Metabolic Roles for Alternative Oxidase in Leaves of Palustrine and Terrestrial Species
Source: Front Plant Sci. 2021 Nov 4;12:752795. doi: 10.3389/fpls.2021.752795 (PMC8600120; doi:10.3389/fpls.2021.752795)
Supplement: Supplementary file 3 [file Table_3.docx]

**Supporting information Table 3.** Modeling of ATP production calculated from values of the *in vivo* activities of cytochrome oxidase (*v*_cyt_) and alternative oxidase (*v*_alt_) in aerial leaves of ten different terrestrial and palustrine plant species. It is considered that 11 ATP are formed for each 6 O_2_ consumed by the AOP and 29 ATP are formed for each 6 O_2_ consumed by the COP (see Materials and Methods). Values are the mean of six to eight measurements obtained from 4-6 plants per species. Different letters indicate significant differences with a p value < 0.05 determined by post hoc Tukey–Kramer's test.

| **Family** | **Habitat** | **Plant species** | ***ATP_cop_***  **(nmol ATP g^−1^ DW s^−1^)** | ***ATP_aop_***  **(nmol ATP g^−1^ DW s^−1^)** | ***ATP_total_***  **(nmol ATP g^−1^ DW s^−1^)** |
| --- | --- | --- | --- | --- | --- |
| Acanthaceae | Palustrine | *Hygrophilla stricta* | 51.92 ± 8.82 **abc** | 10.14 ± 1.44 **bc** | 62.05 ± 10.23 **ab** |
|  | Terrestrial | *Acanthus mollis* | 56.73 ± 5.25 **ab** | 16.58 ± 1.80 **ab** | 73.31 ± 7.02 **a** |
|  |  |  |  |  |  |
| Araceae | Palustrine | *Anubias heterophylla* | 30.63 ± 2.34 **cd** | 3.36 ± 0.53 **d** | 33.99 ± 2.34 **cd** |
|  | Terrestrial | *Arum italicum* | 43.48± 3.50 **bcd** | 13.42 ± 1.28 **abc** | 56.90 ± 4.71 **ab** |
|  |  |  |  |  |  |
| Campanulaceae | Palustrine | *Lobelia cardinalis* | 59.69 ± 7.44 **ab** | 14.73 ± 2.02 **abc** | 74.42 ± 9.25 **a** |
|  | Terrestrial | *Trachelium caeruleum* | 54.32 ± 3.42 **ab** | 17.52 ± 1.50 **a** | 71.84 ± 4.33 **a** |
|  |  |  |  |  |  |
| Polypodiaceae | Palustrine | *Leptochilus pteropus* | 30.01 ± 2.99 **d** | 10.46 ± 1.02 **bc** | 40.48 ± 3.96 **bc** |
|  | Terrestrial | *Polypodium cambricum* | 16.07 ± 1.69 **e** | 9.00 ± 1.07 **cd** | 25.07 ± 2.70 **d** |
|  |  |  |  |  |  |
| Pteridaceae | Palustrine | *Ceratopterys thalictroides* | 78.85 ± 11.36 **a** | 17.95 ± 1.60 **a** | 96.81 ± 12.88 **a** |
|  | Terrestrial | *Pteris vittata* | 31.11 ± 2.80 **cd** | 8.95 ± 0.96 **cd** | 40.06 ± 3.67 **bcd** |
