# Supplementary material for: Different Metabolic Roles for Alternative Oxidase in Leaves of Palustrine and Terrestrial Species
Source: Front Plant Sci. 2021 Nov 4;12:752795. doi: 10.3389/fpls.2021.752795 (PMC8600120; doi:10.3389/fpls.2021.752795)
Supplement: Supplementary file 4 [file Table_4.docx]

**Supporting information Table 4.** Metabolites with higher VIP (variable importance for the projection) values obtained from partial least square sparse regression modeling outputs for each trait. The VIP ranking of metabolites is a representation of the most important metabolites for the models explaining each trait. High VIP values correspond to strong correlations.

|  | ***Palustrine τ_a_*** | | | |  | ***Terrestrial τ_a_*** | |
| --- | --- | --- | --- | --- | --- | --- | --- |
| **Metabolite** | **Comp 1** | **Comp 2** | **Comp 3** | **Comp 4** |  | **Comp 1** | **Comp 2** |
| Phosphoric acid | 2.6 | 2.4 | 2.4 | 2.3 |  | --- | --- |
| Proline | 1.9 | 1.8 | 1.8 | 1.8 |  | --- | --- |
| Malic acid | 1.8 | 1.7 | 1.7 | 1.7 |  | --- | --- |
| Glyceric acid | 1.8 | 1.7 | 1.7 | 1.6 |  | --- | --- |
| Quinic acid | 1.7 | 1.6 | 1.5 | 1.5 |  | --- | --- |
| Caffeoylquinic acid | 1.4 | 1.4 | 1.4 | 1.3 |  | --- | --- |
| Fructose | 1.4 | 1.3 | 1.3 | 1.3 |  | --- | --- |
| GABA | 1.2 | 1.2 | 1.2 | 1.1 |  | --- | --- |
| Threonine | 1.1 | 1.1 | 1.1 | 1.1 |  | --- | --- |
| Glucose | 1.9 | 1.8 | 1.7 | 1.7 |  | 2.4 | 2.2 |
| Trehalose | --- | --- | --- | --- |  | 4.2 | 3.8 |
| Sucrose | --- | --- | --- | --- |  | 2.8 | 2.6 |
| Threonic acid | --- | --- | --- | --- |  | 0 | 2.1 |
| Glycerol | --- | --- | --- | --- |  | 1.5 | 1.3 |
