# Supplementary material for: Different Metabolic Roles for Alternative Oxidase in Leaves of Palustrine and Terrestrial Species
Source: Front Plant Sci. 2021 Nov 4;12:752795. doi: 10.3389/fpls.2021.752795 (PMC8600120; doi:10.3389/fpls.2021.752795)

**Supporting information Figure 1.** Species classification into major biome types of the world, performed from a Whittaker diagram of MAT and MAP, showing the number of records of species from Araceae and Acanthaceae, in which *Higrophylla stricta* (7 records) was substituted for *Higrophylla ringens* (1264 records), and *Anubias heterophylla* (55 records) for *Anubias spp.* Schott. (617 records) for a greater accuracy in the study of species distribution. 1, tropical rainforest; 2, temperate rainforest; 3, tropical seasonal forest; 4, temperate forest; 5, boreal forest; 6, tundra; 7, woodland, shrubland, and grassland; 8, desert.


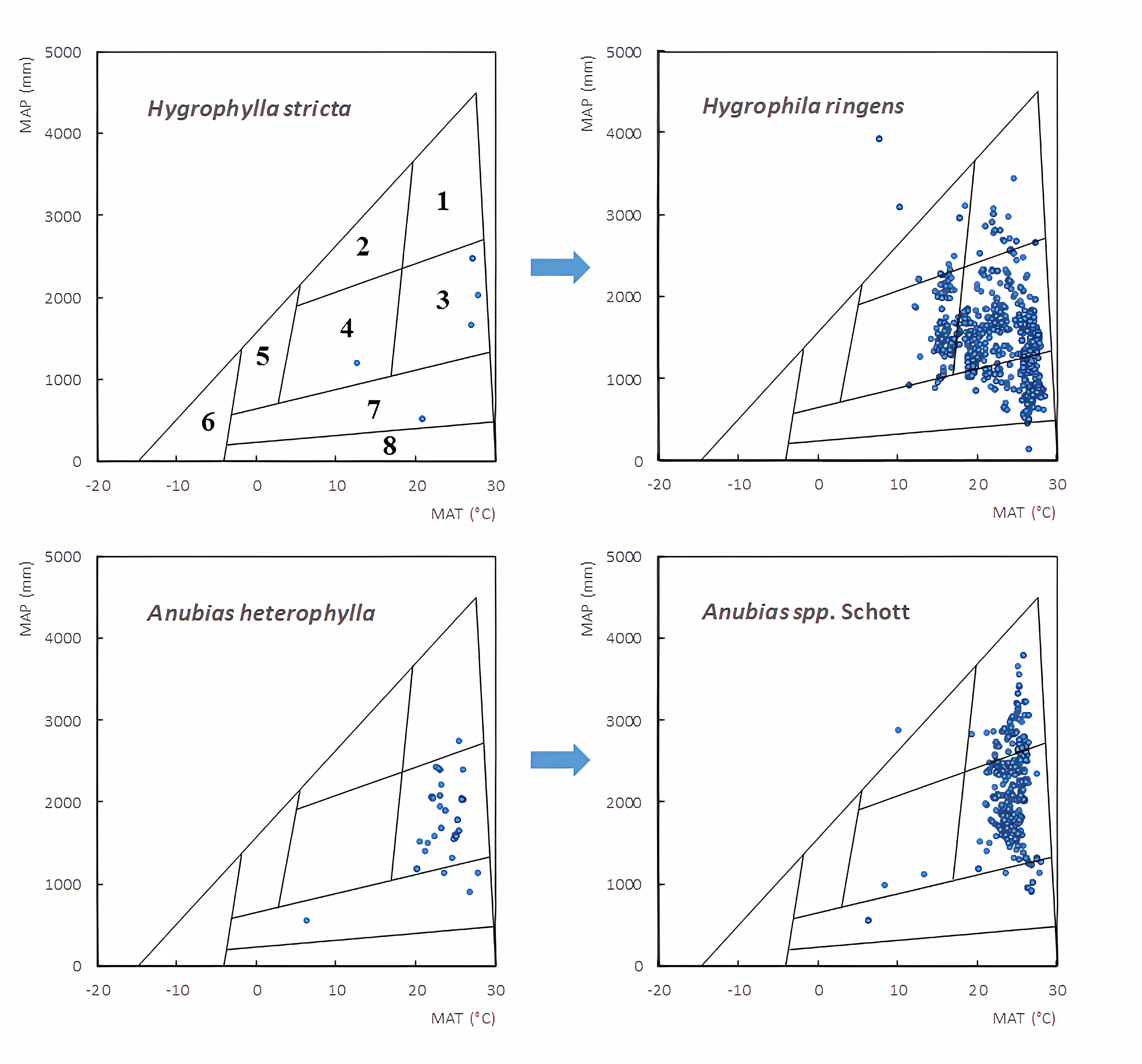

Supplement: Supplementary file 6 [file Data_Sheet_1.docx]
